# Changes in Physiological Indices, Amino Acids, and Volatile Compounds in *Vitis vinifera* L. cv. Pinot Noir under UV-B Radiation and Water Deficit Conditions

**DOI:** 10.3390/foods13040508

**Published:** 2024-02-06

**Authors:** Meng Sun, Yifan Zhu, Brian Jordan, Tao Wang

**Affiliations:** 1 Centre for Viticulture and Oenology, Faculty of Agriculture and Life Sciences, Lincoln University, Christchurch 7647, New Zealand; sm183495665@163.com (M.S.); yifanzhuyy@163.com (Y.Z.); brian.jordan@lincoln.ac.nz (B.J.); 2 Jiangsu Key Laboratory for the Research and Utilization of Plant Resources, Institute of Botany, Jiangsu Province and Chinese Academy of Sciences (Nanjing Botanical Garden Mem. Sun Yat-Sen), Nanjing 210014, China

**Keywords:** UV-B radiation, water deficit, amino acids, aroma compounds, *Vitis vinifera* L. ‘Pinot noir’

## Abstract

UV-B radiation and water deficit can challenge Pinot noir growth and fruit quality. The aim of this work is to determine the effects of UV-B and water deficit on the physiological indices, amino acids, and volatile compounds of Pinot noir vine and fruit. The results showed that both individual and combined treatments caused a decrease in the leaf SPAD, with the largest amplitude being observed in the combined treatment. Water deficit also decreased the leaf water potential and increased the juice δ^13^C‰ at harvest, which was the opposite of the latter under UV-B radiation. Interestingly, most of the physiological indices under combined stresses did not show significant changes compared with that under no UV-B and the well-watered control treatment. Moreover, the concentrations of amino acids and volatile compounds in the berries were determined at harvest. The amino acid contents were significantly increased by the combined treatment, particularly proline (Pro), aspartate (Arg), alanine (Ala), and threonine (Thr). There were slight increases in volatile compounds. This research substantially contributed to improve our scientific understanding of UV-B and water deficit responses in an important commercial species. In addition, it highlighted some future research to produce high-quality wines with the anticipated specific characteristics.

## 1. Introduction

Amino acids and their biosynthesis are important for all living things. Amino acids are the subunits for proteins and enzymes and are also nitrogen and energy sources for yeast and bacterial metabolism [1,2]. In viticulture, amino acids in grapes are precursors of aromatic compounds being metabolised to higher alcohols, aldehydes, organic acids, phenols, and lactones [3]. The total amino acid concentration in grapes increases from veraison to harvest. At harvest, they can account for over 90% of the nitrogen content in musts [4]. The concentration of proline (Pro) in grape juices increases during ripening and reaches a peak pre-harvest; from there, it can slowly reduce until harvest, whereas the aspartate (Arg) concentration rises from veraison to harvest [5,6]. The high concentrations of Pro, threonine (Thr), glycine (Gly), serine (Ser), alanine (Ala), and methionine (Met) in wines are involved in its sweet taste, whereas arginine, lysine (Lys), histidine (His), phenylalanine (Phe), and valine (Val) have a relatively bitter taste. Glutamine (Gln), glutamate (Glu), asparagine (Asn), and Asp have an umami taste [7,8,9]. 

Grape volatile compounds are relevant to grape berries and the quality of wine aroma produced during ripening. These aroma compounds and their precursors of wine quality are established by secondary metabolites during the second growth phase [10]. All volatile grape terpenoids are mono-, sesqui-, or norisoprenoid terpenes produced from the simple isoprene building block, isopentenyl pyrophosphate. Norisoprenoids are commonly derived from carotenoids in plastids and play an important role in the volatile compound make-up of grapes to protect them from oxidative and photo damages [11]. Another group of compounds are found in berry skins and mesocarp: C_6_ compounds (C_6_-aldehydes and C_6_-alcohols) [12]. The C_6_ aldehydes and alcohols can give rise to the characteristic ‘green’ odour, also called ‘green leaf volatiles’ (GLVs). These compounds are induced by the disruption of plant tissues or after plants suffer biotic or abiotic stresses [13].

Ultraviolet solar radiation (UV) is mostly absorbed by the stratospheric ozone layer. Although only 0.5% UV-B (wavelength 280–315 nm) can reach the earth’s ground, it induces the damage of different enzymes and secondary metabolites. The Environmental UV Index is also influenced by the latitude, altitude, season, daytime, and cloud cover [14,15]. Long sunshine hours lead to grapevines’ exposure to more intense UV-B in the regional plantings of grapevines. In addition, the predominant regional plantings of Pinot noir have suffered water deficit during berry development, resulting from reductions in rainfall due to climate change. Thus, the decrease in rainfall has the potential to increase drought risk. Water deficit can cause changes in grapevine metabolism and growth. 

Based on this knowledge, UV-B and water deficit are environmental issues for grapevines in regions. The concentrations and components of amino acids and aroma compounds in grapes are influenced by cultural conditions, rootstock/scion combination, vine management, vineyard location, and growing season [4,16]. However, there has been only very limited research into the effects of a combination of UV-B radiation and water deficit on the chemical composition of grapes. This study contributes to the understanding of the interaction between UV-B radiation exposure, water deficit, and the alteration in physiological traits of the vine and the chemical composition of the fruit in *Vitis vinifera* L. var. Pinot noir. In this study, Pinot noir vines in a glasshouse were subjected to different combinations of UV radiation and water deficit initiated from veraison, and the effects on the physiological status in the vine and the compositions of quality-related compounds in the fruit were investigated. Finally, this research could substantially contribute to improve our scientific understanding of UV-B and water deficit responses in an important commercial species.

## 2. Materials and Methods

### 2.1. Treatments

The Pinot noir vines used in this study were selected from the vineyard located at Lincoln University, Canterbury, New Zealand (43°39′ S, 172°28′ E). These grapevines were moved into a glasshouse in the vineyard for the preparation of experiments in September, prior to budbreak. The glasshouse was made of glass and covered an area of 10 square metres. From October (fruit set) to December (veraison), the grapevines were uniformly irrigated on a regular basis and were exposed to normal daylength hours in the glasshouse. All clusters were harvested in February.

#### 2.1.1. UV-B Treatment

Vines of similar leaf areas and crop weights were divided into two groups of 18 vines. In each group, treatments were applied from veraison to harvest (Table 1) as follows: (i) for UV-B control treatment (−UV), the vines were moved into the glasshouse and no UV-B radiation was applied; (ii) for UV-B treatment (+UV), the vines were put in the same glasshouse, but UV was supplied by UVB-313 UV fluorescent tubes (Q-Lab Company, Westlake, OH, USA). The fluence rates of UV-B (280–313 nm) were measured using a UVB Biometer model 501 radiometer (Solar Light Company, Glenside, PA, USA). A screen hanging between treatments protected the -UV treatments from UV-B radiation. The glasshouse was maintained to the following specifications: 28 °C/18 °C, day/night, and humidity of 70–80%. In the UV-area, the intensity of UV-B was kept at UVI-6 for 8 h/d (9:00–17:00). The relationship between UVI and UV-B intensity is I_UVB_ = 18.9 × UVI (W/m^2^). The expression for UV dose is D = I^4/3^ × t_e_ [(W/m^2^)^4/3^ s], including exposure time (t_e_, s) and UV-B intensity (I, W/m^2^) [17,18,19].

#### 2.1.2. Water Treatment

Vines were exposed to a water treatment in combination with the UV-B treatment. Both UV-B treatment groups were divided into two with two irrigation levels, with each one consisting of nine vines (Table 1). There was a (i) well-watered control treatment where vines were regularly irrigated to soil volumetric water content, reaching around 30% (+W), and a (ii) water deficit treatment where vines received half of the water used in the +W treatment with soil volumetric water content around 10% (−W). Soil in the water deficit treatment was dry to the touch at re-watering, and the grapes had visible shrivelling. Time domain reflectometry (TDR) (Hydrosense™, Campbell Scientific, Inc., Logan, UT, USA) was used to evaluate the percentage of substrate soil moisture for each pot.

#### 2.1.3. Sample Collection

Glasshouse experiments were carried out on potted vines (36 vines) from veraison (12 weeks post bud burst) to harvest (17 weeks post bud burst). Samples from three blocks (3 vines in one block) at 6 weeks post-veraison (harvest) were randomly collected from the control treatment and UV-B or/and water deficit treatments and immediately stored in a walk-in freezer (−20 °C). Sample collection of 10 berries (2 clusters per vine × 1–2 berries per cluster), 20 berries (2 clusters per vine × 3–4 berries per cluster), and 40 berries (2 clusters per vine × 6–7 berries per cluster) from each block were used for the analysis of berry parameters, amino acids, and volatile compositions, respectively. 

### 2.2. Measurement of Physiological Indices in Vines

#### 2.2.1. Leaf Chlorophyll Content

Six fully developed leaves per vine from four replicates at the top, middle, bottom, and both sides of the canopies were randomly selected to be measured for relative chlorophyll contents using a SPAD-502 Plus meter (Konica Minolta Co., Ltd., Osaka, Japan) from veraison to harvest in the 2016–2017 season. All of the values were from six leaf averages to obtain one value per vine. The SPAD value measured the leaf transmittance in two wavelengths: 650 nm and 940 nm [20].

#### 2.2.2. Leaf Water Potential

Leaf water potential (LWP) was determined from one healthy and fully expanded leaf per replicate in the vineyard (one vine in one replicate) and in the glasshouse (one group in one replicate) at harvest in the 2016–2017 season, randomly selected from those close to the clusters. Measurements were performed near solar noon using a pressure chamber (Model 3000; Soil Moisture Equipment Corporation, Santa Barbara, CA, USA).

#### 2.2.3. Time Domain Reflectometry

In the glasshouse, TDR (Hydrosense™, Campbell Scientific, Inc.) was used to evaluate the percentage of substrate soil moisture for each pot and recorded as volumetric water content (%) as a measure of soil water status.

#### 2.2.4. Carbon Isotope Ratio in Leaf Dry Matter and Grape Juice

Six leaves per replicate were randomly collected from the top, middle, and bottom of canopies at harvest and ground to fine powder after freeze-drying. Four milligrams of freeze-dried leaf powder were used for carbon isotope composition measurement. Fifty frozen berries (−20 °C) per replicate were left to stand in a plastic bag at room temperature before the berries were gently crushed using plastic rods to produce grape juice for carbon isotope composition measurement. 

Carbon isotope composition (δ^13^C‰) was analysed by EA-IRMS (Elemental Analyser Isotope Ratio Mass Spectrometry) using a Sercon GSL elemental analyser (Crewe, UK), and a Sercon 20–22 Isotope Ratio Mass Spectrometer (IRMS) (Micromass UK Ltd., Manchester, UK). Samples were analysed in duplicate at a rate of one in eight. δ^13^C‰ was referenced to Vienna-Pee Dee Belemnite standard (V-PDB) and was calculated as proposed by Rao et al. [21]:

(1) $$\delta 13C\left(‰\right)=\frac{Rs-Rb}{Rb}\times 1000$$

where *Rs* is the ^13^C/^12^C ratio of the sample and *Rb* is the ^13^C/^12^C ratio of the PBD Standard.

### 2.3. Chemical Analysis

#### 2.3.1. °Brix, pH, and Titratable Acidity in Grape Juice 

°Brix, TA, and the pH of the grape juice were measured using the method by Bett-Garber et al. [22]: Frozen berries were left to stand in test tubes and defrost to room temperature (20 °C) before processing. The berries were then gently crushed with a plastic rod. A small volume of juice from the berries was used to measure °Brix using a digital refractometer (PAL-1 ATAGO, Tokyo, Japan). 

The rest of the juice was pooled into beakers. Grape juice pH was measured using a Suntex pH/mV/temperature meter (SP-701; Suntex, Taiwan) with a Eutech Instruments probe (EC 620133; Eutech Instruments Pte Ltd., Singapore). Before the analyses, two standard buffer solutions of pH 4.0 and 7.0 were used to calibrate the pH meter. 

Titratable acidity (TA) was determined by titration to pH 8.2 using 0.1 mol/L NaOH (LabServ, 97% min; Biolab (Australia) Ltd., Victoria, Australia). TA was measured on 10 mL of juice for the samples. NaOH (0.1 mol/L) was carefully added into the grape juice under constant stirring using a burette and the volume (mL) used for titration until pH 8.2 was recorded and used for calculations as follows:

(2) TitratableaciditygLasH2T=75×molarity of NaOH×TitrevaluemL÷Volume of juicemL

#### 2.3.2. Amino Acid Analysis

The frozen berries were ground with liquid nitrogen in mortars, transferred into tubes, and centrifuged for 5 min at 1960× *g*. The supernatant was diluted with deionised water (1:4) in a new tube. The grape juice samples were filtered through a 0.45 μmol/L nylon syringe filter into an HPLC glass vial and capped tightly. An internal standard, γ-aminobutyric acid (γ-GABA), was added to a final concentration of 100 μmol/L. For the inline-derivatisation of the primary amino acids, ơ-phthaldialdehyde was used as a fluorescence derivative, iodoacetic acid/mercaptopropionic acid was used to increase cysteine sensitivity, and 9-fluorenylmethyl chloroformate was used as a fluorescence derivative for proline. 

The method of chromatography followed that used by Gregan et al. [23]. The samples were injected into an HPLC system (Hewlett-Packard Agilent 1100 series, Waldbronn, Germany) with a 250 × 4.6 mm, 5 μm Prodigy C18 column (Phenomenex, Milford, MA, USA). Data were analysed using the Chemstation (Agilent) chromatography data system. The mobile phase consisted of two solvents: solvent A (0.01 mol/L Na_2_HPO_4_ with 0.8% tetrahydrofuran, adjusted to pH 7.5 with H_3_PO_4_) and solvent B (20% solvent A, 40% methanol, 40% acetonitrile). The gradient programme was 0 min, 0% B; 14 min, 40% B; 22 min, 55% B; 27 min, 100% B; 35 min, 100% B; and 36 min, 0% B with a flow rate of 1 mL/min. For detection, a fluorescence detector was used with an excitation at 335 nm and emission at 440 nm. At 25 min, the detector was switched to a second channel (excitation at 260 nm and emission at 315 nm) to detect proline. Amino acids were identified by their retention time, and their concentrations were calculated in parallel to calibrate the internal amino acid standard (γ-GABA, 100 μmol/L).

#### 2.3.3. Volatile Compounds Analysis

The analysis of six C_6_ and monoterpene volatile compounds in Pinot noir juice (Table 2) was determined using an automated HS-SPME GCMS (Headspace Solid-Phase Micro-Extraction Gas Chromatograph Mass Spectrometry) technique based on the work by Canuti et al. [24], Dennis et al. [25], Fan et al. [26], Fang and Qian [27], and Yuan and Qian [28]. This adapted method utilised three synthetic deuterated internal standards, namely, hexanal (d_12_) and hexyl (d_13_) alcohol and linalool (d_3_), all obtained from CDN isotopes (Sci Vac Pty Ltd., Montreal, QC, Canada). Eleven non-deuterated standards were used to generate standard curves for quantitative analysis. E-2-Hexenal was obtained from Acros Organics (Sci Vac Pty Ltd., Montreal, QC, Canada), while all other non-deuterated standards were obtained from commercial supplier Sigma-Aldrich (Sci Vac Pty Ltd., Montreal, QC, Canada).

### 2.4. Statistical Analyses

Statistical analysis was undertaken using IBM SPSS Statistics 22. The data were subjected to an independent-sample T-test and two/three-factor analyses (ANOVA) to partition the variance into the main effects (UV-B and water deficit; UV-B, water deficit, and time) and the interaction among them. In the case of significant interactions among factors, treatments were compared using the least significant difference (LSD) at the 5% level (*p* < 0.05).

## 3. Results

### 3.1. Effects of UV-B Radiation Interaction with Water Deficit on Vine Physiology 

**Figure 1:**
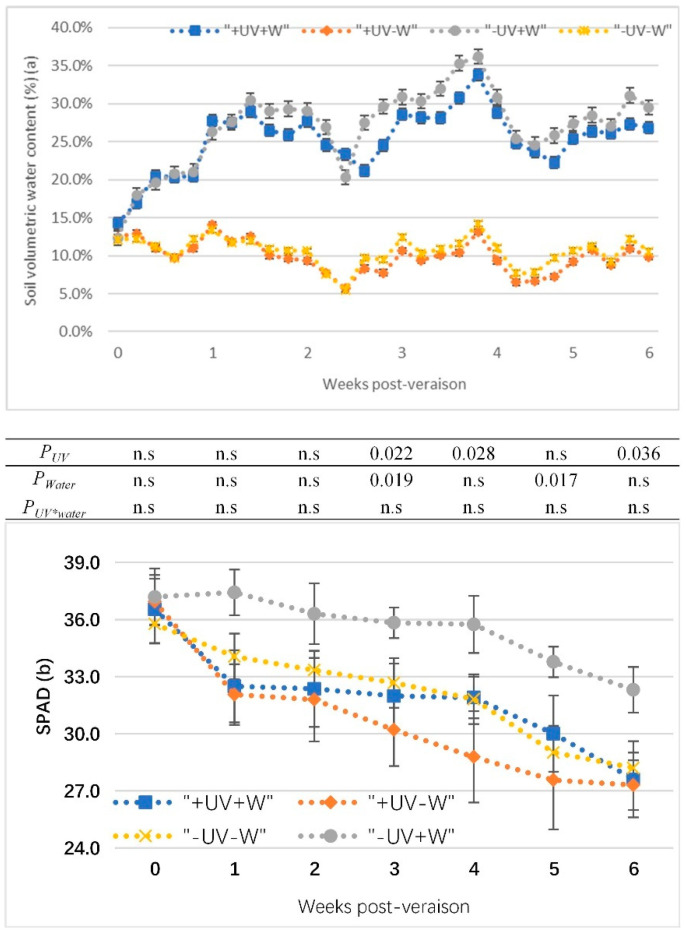
(**a**) The soil volumetric water content of potted vines from veraison to harvest; (**b**) effects of UV-B and water deficit on SPAD level in Pinot noir from veraison to harvest. Data show the means of four replicates. *p*-values are for statistical significance comparing the different treatments according to two-factor ANOVA and LSD test at 5% level; *P_UV_*, UV effects averaged across water treatments; *P_water_*, water effects averaged across UV treatments; P_UV*water_, water effects depend on UV treatments and UV effects depend on water treatments; n.s, no significant difference. +W, well watered, −W, water deficit; +UV, UV-B radiation; −UV, normal light.

The soil volumetric water content of potted vines from veraison to harvest in the glasshouse were presented in Figure 1a. The water deficit treatments (−UV−W and +UV−W) were successful in reducing soil water compared to the well-watered treatments (+UV+W and −UV+W). In +UV−W, the soil volumetric water content was maintained at around 10%, which was about half the value of the soil volumetric water content in the control (−UV+W). 

In all treatments, the leaf SPAD decreased from veraison to harvest (Figure 1b). The SPAD sharply decreased after one week for all treatments, and then it showed a parallel trend. There was no significant difference between the treatments after two weeks of veraison. At 3, 4, and 6 weeks (harvest) post-veraison, under water deficit (−W), +UV−W significantly decreased SPAD compared to −UV−W, while under UV-B (+UV), SPAD had more reduction in +UV−W than +UV+W at 3 and 5 weeks post-veraison.

LWP directly reflected the soil water content and was decreased by water deficit, but not by UV-B (Table 3). Under UV-B stress, the LWP was −1.31 MPa in +UV−W compared to −UV+W at −0.94 MPa and +UV+W at −0.98 MPa. 

UV-B interaction with water deficit (+UV−W) did not influence the carbon isotope ratio in the leaves (Table 3). In the carbon isotope ratio of juice, UV-B caused a drop in the well-watered (+UV+W, −29.16‰) and water deficit (+UV−W, −27.26‰) treatments in comparison with their respective no UV-B treatments (−28.77‰ in −UV+W and −26.75‰ in −UV−W), while the water deficit treatments made the carbon isotope ratio of the juice less negative (Table 3). 

Berry parameters, including °Brix, pH, and TA, were recorded at harvest (Table 3). °Brix was influenced by water treatments. The well-watered treatments had a lower °Brix than the water deficit treatments. A significant difference in TA was shown between the UV treatments, with UV-B causing a decrease. The combination of UV-B and water deficit resulted in a significant difference in pH between treatments. The UV-B treatments significantly increased the pH compared with the no UV-B treatments. +UV−W caused a smaller increase in pH than +UV+W. 

### 3.2. Effects of UV-B Radiation Interaction with Water Deficit on Amino Acids

The concentration of amino acids was changed in response to UV-B and water deficit, except for Cys and Met (Table 4). The total amino acid concentration was increased by UV-B or water deficit, and the effects of the individual stress were qualified by the UV-B interaction with water deficit. The concentration of total amino acids was higher in the water deficit treatments than in the well-watered treatments, but the effect of water depended on the UV-B conditions. Overall, the total amino acid concentration was the highest at 4150 µM in +UV−W than in the other treatments. Also, the combination of UV-B and water deficit changed the concentration of the other amino acids. The most abundant amino acids were Pro, Arg, and Ala, reaching over 300 µM in the treatments. The concentrations of Pro and Ala under water deficit were higher under +UV than under −UV. There were also higher Pro and Ala concentrations under +UV+W than under −UV+W. Arg was increased by 2% in +UV+W and by 4% in −UV−W compared to −UV+W. +UV−W enhanced an increase in Arg (1357 µM), which was double the concentration in −UV+W (608 µM).

The individual amino acid percentages showed significant changes in the berries exposed to UV-B and water deficit together (Table 5). The α-ketoglutarate family accounted for the highest proportion of amino acids, but in terms of the treatment effects, there was only an interaction between UV-B and water deficit on Arg. When the vines were undergoing the well-watered treatment, the percentage of Arg was larger under no UV-B than with UV-B. However, the value under −UV−W was 28% lower than 33% +UV−W. Other amino acid percentages in the shikimate (aromatic) and aspartate families were affected by the combined stresses but accounted for low percentages of the total. 

### 3.3. Volatile Compounds

The combination of UV-B and water deficit induced significant changes in hexanol, (E)-2-hexenol, and nerol. The vines under water deficit had higher concentrations of hexanol and (E)-2-hexenol in the UV-B treatment than in the no UV-B treatment (Table 6). The concentrations of hexanol and (E)-2-hexenol in +UV−W had values of 1029 µg/L and 790 µg/L, respectively, compared to +UV+W at 752 µg/L and 530 µg/L, respectively. The nerol concentration in −UV+W was 3.4 µg/L, which was the highest of any of the treatments. 

## 4. Discussion

Water deficit can dramatically influence UV-B-induced responses, but the responses from water deficit and UV-B depended on the plant species. The interactions between UV-B exposure and water deficit in plants have been investigated for about 30 years, but few have been undertaken about the effects in Pinot noir. In this study, we investigated the effects of UV-B interaction with water deficit on the physiological statuses of the vine and amino acids and the volatile compositions of the fruit.

### 4.1. The Alteration in Vine Physiological Indices Induced by UV-B Interaction with Water Deficit 

The combination of UV-B and water deficit caused decreases in the leaf water potential (LWP) (Table 3). Compared to either individual stress, the combination of UV-B and water deficit did not increase the magnitude of the responses. Therefore, the combination of UV-B and water deficit decreased LWP induced by water deficit alone (−1.31MPa in +UV−W and −1.38 MPa in −UV−W) [29,30,31]. 

There was no interaction effect of UV-B and water deficit on the SPAD levels (Figure 1b), but the statistical analyses showed a UV effect averaged across water treatments (+UV+W and +UV−W vs. −UV+W and −UV−W), as well as significant differences in water deficit. UV-B decreased the SPAD value during ripening compared with the control (Figure 1b), which was consistent with the observations by Núñez-Olivera et al. [32]. The environmental parameters (temperature and humidity) in the glasshouse were controlled by a thermostat during the trial period, so grapevines in the control and the UV-B treatment received the exact same temperature over the whole trial period. The two layers of 125-micron clear natural polythene laid over the top of the glass resulted in a reduction of approximately 66% PAR and the exclusion of UV-A/B. The low ratio of PAR to UV-B may enhance the sensitivity of leaves to UV-B [33] and may show changes in the pigment composition and stomatal resistance [34]. It may be that the SPAD value depends on the light transmittance of leaves, where decreases in the SPAD value are associated with increases in light transmittance [35]. UV-B leads the chloroplasts to move to the periclinal cell walls with an increase in light transmittance as a result, which could be associated with a decrease in SPAD in the glasshouse. It was assumed that the combined stresses can lead to enhanced light transmittance through leaves, resulting in a decrease in SPAD when compared to the individual treatments. However, the final results showed that the combination of UV-B and water deficit did not change the response compared to either UV-B or water deficit.

As with water, carbon dioxide (CO_2_) is another important compound for the synthesis of carbohydrate through photosynthesis. So, taking measurements of the abundant stable isotope carbon atoms in plant tissues is a way to evaluate the effects of UV-B and water deficit on grapevines [36]. We found that water deficit or UV-B radiation caused increased or decreased juice δ^13^C‰, and no significant changes in the leaf and juice δ^13^C‰ were measured in the combined treatment (Table 3). In grapevine tissues, the ^13^C/^12^C isotope ratio is determined by the gradient of CO_2_ concentrations between the atmospheric and intercellular spaces in the leaf, which is influenced by environmental stresses [37,38]. UV-B radiation decreases the activity of Rubisco [39]. Stomatal closure leads to less diffusion and then decreases CO_2_ uptake in grapevine leaves induced by water deficit. Thus, intercellular ^13^CO_2_ is more likely to be used as the substrate of Rubisco in the carboxylation reaction. Grapevines grown under water stress, such as Merlot, Cabernet Sauvignon, Cabernet franc, and Tempranillo, tend to have more positive carbon isotope ratios (δ^13^C‰) [37,40]. So, the results stated that a water deficit could make up UV-B-induced responses in vine carbon isotope assimilation. More severe stress and more restricted stomata openings lead to photosynthates with a greater proportion of ^13^C [41]. From source to sink, most sucrose (photosynthates) containing ^13^C is translocated from the leaves to grapes and converted to fructose and glucose, resulting in an increased ^13^C in fructose and glucose [42,43]. Additionally, less photosynthates incorporating ^13^C in leaves are used to maintain function, such as respiration [41]. Thus, UV-B or water deficit could affect the δ^13^C‰ of juice in an opposite way, but their combination did not show significant effects on the leaves or juice δ^13^C‰.

### 4.2. Effects of UV-B Interaction with Water Deficit on Amino Acids in Berries

There is little research about the effects of UV-B interaction with water deficit on amino acids in grapes. UV-B radiation in combination with water deficit increased the concentrations of total amino acids and some individual amino acids (Table 4 and Table 5). There was a higher concentration of free Arg than Pro in the grapes at harvest, because Pinot noir is an Arg-accumulating cultivar [44]. The increases in amino acids appeared to be from water deficit, increasing the concentration of total free amino acids due to increases in some individual amino acids of berries, particularly Pro, Arg, Ala, and Thr [45]. Pro and Arg are major components of total amino acids in grapes and can function for an osmotic adjustment and act as antioxidants [46]. Pro biosynthesis is a reductive pathway controlled by the activation of Δ1-pyrroline-5-carboxylate synthetase (*P5CS*) and Δ1-pyrroline-5-carboxylate reductase (*P5CR*) genes and requires NADPH. A water deficit induces the accumulation of NADPH due to the inhibition of the Calvin cycle, so the accumulation of Pro under water deficit generates NADP^+^ and maintains a low NADPH: NADP^+^ ratio for the Calvin cycle [47,48]. UV-B may enhance the increases in Pro and Arg under water deficit. UV-B, in combination with a water deficit, could induce greater degradation of proteins to produce more amino acids for osmotic adjustment [49,50]. Therefore, the combination of UV-B and water deficit can increase the concentration of total amino acids, particularly Arg and Pro. 

### 4.3. The Effects of UV-B Interaction with Water Deficit on Volatile Composition in Berry Juice

The C_6_ aldehydes, C_6_ alcohols, and monoterpenes are the most important volatile compounds responsible for the varietal aromas of grapes and wines [51]. To investigate the effects of UV-B radiation interacting with water deficit on volatile compounds in Pinot noir juice, samples were taken at harvest (Table 6). In the berry skin of mesocarps, C_6_ compounds (C_6_-aldehydes and C_6_-alcohols) are formed by the enzymatic oxidation of unsaturated lipids during ripening. Some alterations in the C_6_ compounds in berries may be explained by UV-B causing an increase in the abundance of transcripts of several lipoxygenases (LOX) in grapevines to produce a cascade of damaging ROS, resulting in the increased catabolism of fatty acids to C_6_ compounds [52,53]. Water deficit causes an increase in the transcript abundance of LOX and hydroperoxide lyase (HPL), resulting in the increase in the conversion from the hydroperoxides to volatile esters [54,55]. However, it also increases the transcript abundance of alcohol dehydrogenase (ADH) and alcohol acyltransferase (AAT), which catalyses the branched-chain alcohols derived from the metabolism of amino acids to form volatile esters [56]. Therefore, UV-B interaction with water deficit potentially enhanced the abundance of transcripts of LOX, HPL, and ADH to produce C_6_ alcohols (hexanol and (Z)-3-hexenol) compared to either UV-B or water deficit alone.

## 5. Conclusions

In this study, the changes in physiological indices, amino acids, and volatile compounds in Pinot noir were shown in response to UV-B or water deficit and their combination. Individual or combined treatments decreased the SPAD and leaf water potential with varying degrees, which potentially affected the physiological growth of vines. However, in terms of CO_2_ utilisation, UV-B and water deficit did not show significant changes in the leaf δ^13^C‰, but caused opposite effects on the juice δ^13^C‰, while this phenomenon was offset by UV-B in combination with water deficit. Moreover, effective increases in amino acids under UV-B interaction with water deficit may act as the precursors of volatile compounds in grapes, resulting in significant effects on the aroma and flavour characteristics of wine. Overall, this study investigated the effects of water and UV-B on grape growth and quality, but the results are partial and require further studies. In addition, the changes in the temperature and CO_2_ under the frames were not controlled, which are also key issues for global grapevine and wine production, and they should be considered in future research.

## Figures and Tables

**Table 1:** Glasshouse treatments (three vines in a block).

Water Treatment	UV-B Treatment	Natural Light
Well watered	+W+UV (9 vines)	+W−UV (9 vines)
Water deficit	−W+UV (9 vines)	−W−UV (9 vines)

**Table 2:** Deuterated and non-deuterated standards for six C_6_ and five monoterpene volatile compounds in Pinot noir juice.

Compound	ISTD ID No	RT (min)	Target Ion (*m*/*z*)	Confirming Ions (*m*/*z*, % of Target)	Calibration Range ^ (µg/L)	CAS No.
d_12_ hexanal	ISTD 1	7.78	64	48 (140.2), 46 (92.6)	-	1219803-74-3
n-Hexyl d_13_ Alcohol	ISTD 2	10.12	64	50 (45.2), 46 (44.1)	-	16416-34-5
d_3_ linalool	ISTD 3	12.31	96	124 (25.9), 139 (10.1), 58 (16.8)	-	1216673-02-7
Hexanal	1	7.85	44	41 (77.8), 56 (75.2)	0–1048.6	66-25-1
(E)-2-Hexenal	2	9.23	41	55 (74.4), 39 (59.5)	0–1517.1	6728-26-3
1-Hexanol	2	10.26	56	43 (64.5), 55 (51.3)	0–824.1	111-27-3
(E)-3-Hexen-1-ol	2	10.33	67	82 (58.1), 100 (3.8)	0–23.4	928-97-2
(Z)-3-Hexen-1-ol	2	10.53	41	67 (78.2), 55 (38.8)	0–265.4	928-96-1
(E)-2-Hexen-1-ol	2	10.70	57	41 (50), 39 (20.5)	0–513.3	928-95-0
Linalool	3	12.35	93	12 (28.0), 136 (8.8)	0–8.6	78-70-6
Citronellol	3	15.44	138	82 (468.2), 95 (397.3), 109 (138.2)	0–8.2	7540-51-4
α-terpineol	3	14.59	93	121 (75.8), 136 (60.9), 81 (61.36)	0–6.3	10482-56-1
Nerol	3	16.06	68	123 (28.9), 139 (18.1), 136 (11.4)	0–7.3	106-25-2
Geraniol	3	16.88	84	93 (122.3), 123 (98.9)	0–13.3	106-24-1

^ All samples were diluted 2-fold with 0.2 mol/L citrate buffer; hence, concentrations obtained were multiplied by this factor accordingly.

**Table 3:** Effects of UV-B and water deficit on leaf water potential; δ^13^C‰ of leaf and juice and berry parameters in Pinot noir at harvest.

Physiological Indices	+UV+W	+UV−W	−UV+W	−UV−W	*P_UV_*	*P_water_*	P_UV*water_
Leaf water potential (MPa)	−0.98	−1.31	−0.94	−1.38	n.s	<0.001	n.s
Leaf ^13^C vs. V-PDB ‰	−28.27	−28.88	−29.07	−28.36	n.s	n.s	n.s
Juice ^13^C vs. V-PDB ‰	−29.16	−27.26	−28.77	−26.75	0.001	<0.001	n.s
°Brix	21.0	21.0	20.1	21.7	n.s	0.010	n.s
TA (g/L)	6.1	6.6	7.3	7.5	<0.001	n.s	n.s
pH	3.41	3.30	3.23	3.23	0.001	n.s	0.040

Data show the means of three replicates from harvest in the 2016–2017 period. *p*-values for statistical significance comparing the different treatments according to two-factor ANOVA and LSD test at 5% level. *P_UV_*, UV effects averaged across water treatments; *P_water_*, water effects averaged across UV treatments; P_UV*water_, water effects depend on UV treatments and UV effects depend on water treatments; n.s, no significant difference. +W, well-watered; −W, water deficit; +UV, UV-B radiation; −UV, normal light.

**Table 4:** Effects of UV-B and water deficit on amino acids in Pinot noir berries at harvest.

Amino Acid (µM)	+UV+W	+UV−W	−UV+W	−UV−W	*P_UV_*	*P_water_*	P_UV*water_
α-ketoglutarate							
Pro	487	825	321	511	0.001	0.001	0.007
Arg	622	1357	608	629	<0.001	<0.001	<0.001
Glu	100	141	100	99	<0.001	<0.001	<0.001
Gln	45	91	43	56	<0.001	<0.001	<0.001
His	16	37	11	15	<0.001	<0.001	<0.001
Shikimate (aromatic)							
Phe	17	52	18	19	<0.001	<0.001	<0.001
Trp	13	82	13	27	<0.001	<0.001	<0.001
Tyr	7	15	5	8	<0.001	<0.001	<0.001
Pyruvate							
Leu	33	87	25	31	<0.001	<0.001	<0.001
Val	17	69	20	45	0.031	<0.001	0.009
Ala	336	624	297	357	<0.001	<0.001	<0.001
Aspartate							
Asp	54	59	51	50	<0.001	n.s	0.026
Asn	15	33	13	19	<0.001	<0.001	<0.001
Thr	161	327	176	183	<0.001	<0.001	<0.001
Ile	25	86	20	28	<0.001	<0.001	<0.001
Met	5	8	4	11	n.s	0.002	n.s
Lys	19	28	18	17	<0.001	0.002	0.001
3-phosphoglycerate							
Cys	2	1	3	4	n.s	n.s	n.s
Ser	116	207	113	130	<0.001	<0.001	<0.001
Gly	12	22	10	13	<0.001	<0.001	<0.001
Total	2101	4150	1869	2254	<0.001	<0.001	<0.001

Data show the means of three replicates from harvest in the 2016–2017 period. *p*-values for statistical significance comparing the different treatments according to two-factor ANOVA and LSD test at 5% level. *P_UV_*, UV effects averaged across water treatments; *P_water_*, water effects averaged across UV treatments; P_UV*water_, water effects depend on UV treatments and UV effects depend on water treatments; n.s, no significant difference. +W, well-watered; −W, water deficit; +UV, UV-B radiation; −UV, normal light.

**Table 5:** Effects of UV-B and water deficit on the percentages of each amino acid in total amino acids in Pinot noir berries at harvest.

	+UV+W	+UV−W	−UV+W	−UV−W	*P_UV_*	*P_water_*	P_UV*water_
α-ketoglutarate							
Pro	23.2%	19.9%	17.2%	22.7%	n.s	n.s	n.s
Arg	29.6%	32.7%	32.5%	27.9%	n.s	n.s	0.008
Glu	4.8%	3.4%	5.4%	4.4%	<0.001	<0.001	n.s
Gln	2.1%	2.2%	2.3%	2.5%	0.001	n.s	n.s
His	0.7%	0.9%	0.6%	0.7%	<0.001	0.001	n.s
Shikimate (aromatic)							
Phe	0.8%	1.3%	1.0%	0.8%	0.002	<0.001	<0.001
Trp	0.6%	2.0%	0.7%	1.2%	<0.001	<0.001	<0.001
Tyr	0.3%	0.4%	0.3%	0.4%	n.s	0.003	0.024
Pyruvate							
Leu	1.6%	2.1%	1.3%	1.4%	<0.001	<0.001	0.002
Val	0.8%	1.7%	1.1%	2.0%	n.s	0.001	n.s
Ala	16.0%	15.0%	15.9%	15.8%	n.s	n.s	n.s
Aspartate							
Asp	2.6%	1.4%	2.7%	2.2%	<0.001	<0.001	<0.001
Asn	0.7%	0.8%	0.7%	0.9%	n.s	<0.001	0.020
Thr	7.7%	7.9%	9.4%	8.1%	<0.001	0.016	0.003
Ile	1.2%	2.1%	1.1%	1.3%	<0.001	<0.001	<0.001
Met	0.2%	0.2%	0.2%	0.5%	0.039	n.s	0.021
Lys	0.9%	0.7%	1.0%	0.8%	n.s	0.001	n.s
3-phosphoglycerate							
Cys	0.1%	0.0%	0.2%	0.2%	0.010	n.s	n.s
Ser	5.5%	5.0%	6.0%	5.8%	<0.001	<0.001	n.s
Gly	0.6%	0.5%	0.5%	0.6%	n.s	n.s	n.s

Data show the means of three replicates from harvest in 2016–2017 period. *p*-values for statistical significance comparing the different treatments according to two-factor ANOVA and LSD test at 5% level. *P_UV_*, UV effects averaged across water treatments; *P_water_*, water effects averaged across UV treatments; P_UV*water_, water effects depend on UV treatments and UV effects depend on water treatments; n.s, no significant difference. +W, well-watered; −W, water deficit; +UV, UV-B radiation; −UV, normal light.

**Table 6:** Effects of UV-B and water deficit on volatile compounds in Pinot noir juice at harvest.

Volatile Compounds (µg/L)	+UV+W	+UV−W	−UV+W	−UV−W	*P_UV_*	*P_water_*	P_UV*water_
C_6_ aldehydes							
Hexanal	223.1	50.6	193.5	169.0	n.s	0.044	n.s
(E)-2-hexenal	119.6	79.9	128.8	96.7	n.s	0.016	n.s
C_6_ alcohols							
Hexanol	751.8	1028.6	683.3	646.2	0.005	n.s	0.029
(E)-3-hexenol	11.9	12.7	11.2	11.8	n.s	n.s	n.s
(Z)-3-hexenol	29.3	26.6	30.0	29.6	n.s	n.s	n.s
(E)-2-hexenol	529.5	790.3	571.3	562.2	n.s	0.031	0.023
Free monoterpenes							
Linalool	1.5	1.5	1.5	1.5	n.s	n.s	n.s
α-terpineol	1.0	1.1	1.2	1.2	n.s	n.s	n.s
Citronellol	1.0	1.2	1.1	1.1	n.s	0.037	n.s
Nerol	3.0	3.0	2.7	3.4	n.s	n.s	0.026
Geraniol	13.5	13.3	13.3	14.3	n.s	n.s	n.s

Data show the means of three replicates from harvest in 2016–2017 period. *p*-values for statistical significance comparing the different treatments according to two-factor ANOVA and LSD test at 5% level. *P_UV_*, UV effects averaged across water treatments; *P_water_*, water effects averaged across UV treatments; P_UV*water_, water effects depend on UV treatments and UV effects depend on water treatments; n.s, no significant difference. +W, well-watered; −W, water deficit; +UV, UV-B radiation; −UV, normal light.

## Data Availability

The data presented in this study are available on request from the corresponding author.
